# Time-Lapse 3D CSEM for Reservoir Monitoring Based on Rock Physics Simulation of the Wisting Oil Field Offshore Norway

**DOI:** 10.3390/s23167197

**Published:** 2023-08-16

**Authors:** Mohammed Ettayebi, Shunguo Wang, Martin Landrø

**Affiliations:** Department of Electronic Systems, Norwegian University of Science and Technology, NO-7491 Trondheim, Norway; shunguo.wang@ntnu.no (S.W.); martin.landro@ntnu.no (M.L.)

**Keywords:** marine CSEM, 3D time-lapse, reservoir monitoring, production simulations

## Abstract

The marine controlled-source electromagnetic (CSEM) method has been used in different applications, such as oil and gas reservoir exploration, groundwater investigation, seawater intrusion studies and deep-sea mineral exploration. Recently, the utilization of the marine CSEM method has shifted from petroleum exploration to active monitoring due to increased environmental concerns related to hydrocarbon production. In this study, we utilize the various dynamic reservoir properties available through reservoir simulation of the Wisting field in the Norwegian part of the Barents Sea. In detail, we first developed geologically consistent rock physics models corresponding to reservoirs at different production phases, and then transformed them into resistivity models. The constructed resistivity models pertaining to different production phases can be used as input models for a finite difference time domain (FDTD) forward modeling workflow to simulate EM responses. This synthetic CSEM data can be studied and analyzed in the light of production-induced changes in the reservoir at different production phases. Our results demonstrate the ability of CSEM data to detect and capture production-induced changes in the fluid content of a producing hydrocarbon reservoir. The anomalous CSEM responses correlating to the reservoir resistivity change increase with the advance of the production phase, and a similar result is shown in anomalous transverse resistance (ATR) maps derived from the constructed resistivity models. Moreover, the responses at 30 Hz with a 3000 m offset resulted in the most pronounced anomalies at the Wisting reservoir. Hence, the method can effectively be used for production-monitoring purposes.

## 1. Introduction

The marine controlled-source electromagnetic (CSEM) method has been applied in different applications, such as hydrocarbon and hydrates exploration [1,2], drilling hazards mitigation [3,4], sub-basalt or sub-salt imaging [5,6], freshwater investigations [7,8], and lithosphere and crust studies [9,10,11]. CSEM is widely acknowledged as an efficient method in exploration due to its high sensitivity in distinguishing resistive and conductive anomalies, such as oil and brine reservoirs [10]. Recently, several studies have been devoted to extending the applications of the CSEM method from being a risk-reduction tool to a time-lapse production-monitoring tool, such as petroleum production [12,13,14] and CO_2_ storage monitoring [15,16]. Since CSEM data have sensitivity to pore fluid saturation, time-lapse CSEM can offer valuable information, especially when time-lapse seismic methods fail to perform effective monitoring. For example, seismic methods tend to not clearly demarcate the response difference between pore pressure and saturation changes [17]. However, time-lapse CSEM can provide us with information that closes this gap [18,19], and thus CSEM and seismic data can complement each other. In 2009, Zach et al. and Orange et al. [12,20] presented the possibility of extending CSEM applications from being a mere exploration tool to also a time-lapse-monitoring and reservoir-management tool. Increased attention is also drawn toward understanding the time-lapse behavior of the CSEM method in more detail [21,22,23]. However, there is still a lack of research that thoroughly studies the potential of the CSEM method in capturing production-induced changes in the fluid content for realistic and detailed reservoir models at different production phases. Most of the work carried out on time-lapse CSEM does not address realistic and complex fluid flow simulations of the producing reservoir [24,25]. There is also no established methodology to process time-lapse CSEM data [26]. Therefore, we study how to enable time-lapse 3D CSEM for reservoir monitoring based on knowledge of the Wisting reservoir.

**Figure 1:**
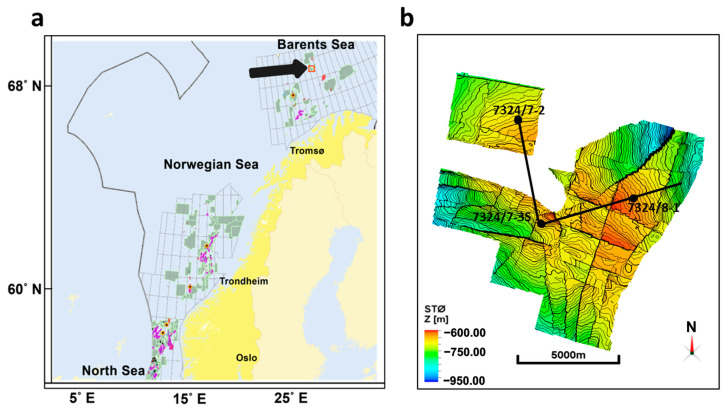
The Wisting field. (**a**) Geographical map of Wisting, offshore Norway, showing that the field is located on the Norwegian part of the Barents Sea as highlighted by the red rectangle. On this map, hydrocarbons are indicated by both red and magenta, the lightish green highlights different production licenses and yellow and blue are for the basemaps. This subfigure is modified after Norwegian Petroleum Directorate (NPD) FactPages. (**b**) The structure depth map of the Stø formation, which is one of the main formations of the reservoir. The thick black lines are intersection lines going through specific wells marked by the black dots. The colors highlight the depth of the horizon below the sea surface. This subfigure was generated using Petrel 2019.4.

**Figure 2:**
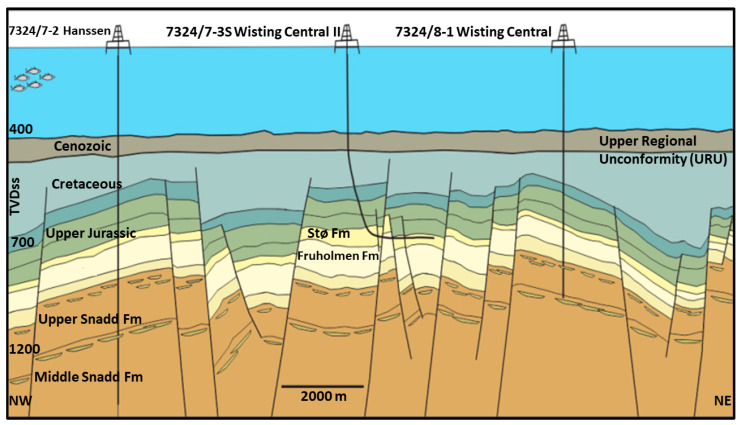
Schematic representation of the local structural and sedimentological geology along both of the thick black lines shown in Figure 1b. The Nordmela Formation is located between Stø and Fruholmen formations. However, it is not shown here since it is too thin to be resolved in this figure. This figure is modified after Stueland [30].

The Wisting reservoir is in the Norwegian part of the Barents Sea (Figure 1a), and the Wisting discovery was made back in 2013 by the wildcat well 7324/8-1 (Figure 1b). The well is drilled-down flanks on a structure in the Wisting Central prospect and hence given the name of central well. The reservoir in this area is of good quality, and belongs to the Realgrunnen Subgroup which consists of three formations: Fruholmen, Nordmela and Stø formations (Figure 2). The central well (7324/8-1) was drilled with the primary objective of investigating and evaluating the Jurassic Realgrunnen Subgroup for hydrocarbons [27]. The well enters the top of the reservoir which is the Stø formation at a depth of about 650 m. The water depth at the site is 400 m and the reservoir top is currently buried 250 m beneath the seafloor. Wisting is known for its discernible electric properties where the background resistivity is high and can reach 20 Ωm [28,29]. Due to the shallow burial depth, typical marine CSEM signals (0.1–16 Hz) have the desired penetration depths to the reservoir.

The assumed recovery mechanism for the production strategy utilized for Wisting in this study is oil production with water injection to improve recovery and delivery. All production and injection wells are planned to be horizontal. The field is compartmentalized, and each major, hydrocarbon-bearing fault segment is planned to be developed by one or more pairs of production and injection wells. The injection will be radial around the well completion grid blocks, assuming the grid properties in the surrounding cells have sufficient transmissibility. Therefore, the injection flow pattern around the well will depend on parameters like permeability, grid cell geometry, and transmissibility modifiers due to, e.g., fault and well geometries.

With different geophysical studies at the site and planned details for production, a rich database is available for this time-lapse study, consisting of eclipse reservoir flow simulations, well logs, 3D-CSEM inversion results and both conventional and P-cable 3D seismic data. The seismic data have been used by the company Österreichische Mineralölverwaltung Aktiengesellschaft (OMV) in making the geological structure of the reservoir model. The company then provided us with dynamic reservoir flow simulation models of the Wisting field incorporating different properties, such as porosity, water and oil saturation, and permeabilities for each time step at different production phases. We then employed and combined the available data to various extents into a rock physics framework that enabled us to create a series of realistic resistivity models to represent different oil production phases. Subsequently, the resistivity models were utilized to model CSEM data, and a finite difference time domain (FDTD) software, SBLwiz developed by EMGS [31], was used to fulfill the modeling at different production phases. Then, we used the generated resistivity models and modeled data to investigate the survey reliability and feasibility of effective and safe production monitoring using time-lapse CSEM.

Therefore, this study consists of two main parts. The first one deals with the construction and calibration of the reservoir resistivity models while the second is devoted to studying the reliability and feasibility of time-lapse CSEM for oil production monitoring.

## 2. Method

### 2.1. The Marine CSEM Method

**Figure 3:**
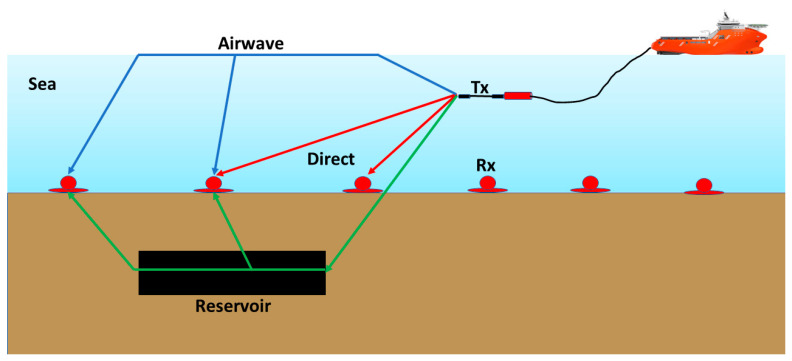
The main principles of marine CSEM for hydrocarbon exploration are presented, where the transmitter *Tx* is an HED that excites an electric pulse. The direct waves are indicated by red. The blue color highlights the airwave, and the green is for the field component that propagates through the thin resistor and back to the receivers *Rx*.

Marine CSEM observes EM fields on/above the seafloor and images subsurface structures through electrical resistivity models. It requires both a transmitter and multiple receivers. The receivers can either be deployed on the seafloor or be towed behind the transmitter above the seafloor. Being able to detect and image high resistivity anomalies of saturated petroleum reservoirs, the marine CSEM method has gained increasing popularity in the oil industry, and hence its application since the first publication that demonstrated the concept in 2002 [32]. The source is typically a high current horizontal electric dipole (HED), and the current strength of the newly developed powerful transmitters can reach 10,000 A. The transmitting frequency typically used is from 0.1 to 30 Hz. The HED is ideally towed approximately 30 m above the seabed. This is because one wants the transmitter to be as close as possible to the seafloor to maximize interaction with seafloor rocks and sediments without damaging it by being too close to the seafloor [1]. The standstill receivers are deployed on the seafloor with spacing distances ranging from 1 to 5 km according to the survey objective and layout (Figure 3). The receivers can be deployed along a line, resulting in a two-dimensional (2D) survey, or along multiple lines or around a 2D grid when 3D resistivity imaging is required. The receivers can measure the electromagnetic fields that interact with seafloor structures.

The Wisting field has demonstrated outstanding CSEM sensitivity [29] due to a combination of high background resistivities and a shallow reservoir burial depth. This has resulted in several CSEM surveys being carried out. The latest one was in 2014 with a denser data coverage and higher frequencies than the earlier surveys from 2008 and 2013. The inversion results were produced by an industrial partner and are available to us for petrophysical model calibration at the assumed beginning of the production phase.

### 2.2. Anomalous Transverse Resistance

Obtaining insight into the different rock properties is of critical importance for CSEM time-lapse studies. Especially, constructing a realistic rock physics model that can be used for time-lapse CSEM studies will depend on the different properties of the reservoir, such as porosity, fluids in the pores, resistivities of fluids and rocks, etc. This unavoidably requires precise and realistic mapping. Because resistivity models alone cannot achieve this goal, sometimes due to model uncertainties, anomalous transverse resistance (ATR) is then applied. The CSEM response of a hydrocarbon reservoir is determined by its ATR. ATR is an attribute that was first introduced to circumvent the challenge of masked reservoirs by complex resistivity backgrounds [33]. The primary utility of ATR in the context of time-lapse CSEM is to relieve the ambiguities of the resistivity model due to geometrical uncertainty related to the low frequencies employed in the CSEM surveys. ATR represents the intrinsic earth property and is defined as the integral of resistivity over the thickness of the reservoir,

(1) $$ATR=\sum \Delta z_{i}(R_{i}-R_{i}^{Background}).$$

where Δ*z_i_* represents the thickness of the ith model layer/cell and *R_i_* represents the resistivity of the ith layer/cell. *R_i_^Background^* represents the background resistivity surrounding the reservoir. Equation (1) defines the ATR laterally in terms of resistivity and thickness while background resistivity is defined as the water-saturated reservoir resistivity. Using Equation (1), varying resistivities can be transformed to ATR maps [23,29] and hence reduce the uncertainties of the reservoir properties. 

## 3. Resistivity Model Construction

### 3.1. Petrophysical Model Selection

The reservoir resistivity depends on several subsurface parameters, such as fluid content in the pore space and its resistivity, fluid saturation, matrix resistivity, etc. Archie’s equation relates electrical resistivity to all those parameters; therefore, it is widely used to further interpret electrical resistivity models inverted from CSEM data. Equation (2) represents Archie’s equation [34]:

(2) $$\frac{1}{R_{t}}=\frac{\varphi^{m}S_{W}^{n}}{a\;R_{W}}.$$

Here, *S_w_* is the brine saturation in the pore space, *a* refers to the formation tortuosity factor and is usually set equal to unity [35], *R_w_* is water resistivity at formation temperature, *φ* is porosity, *R_t_* is formation resistivity, *n* is the saturation exponent, and *m* is the cementation factor. Archie’s law assumes that the only conductive material is brine and that the rock matrix is non-conductive [36]. However, if the reservoir contains clay, then more advanced models are required to represent it. We will show that this is not our case in the next subsection.

### 3.2. Petrophysical Parameter Selection

**Figure 4:**
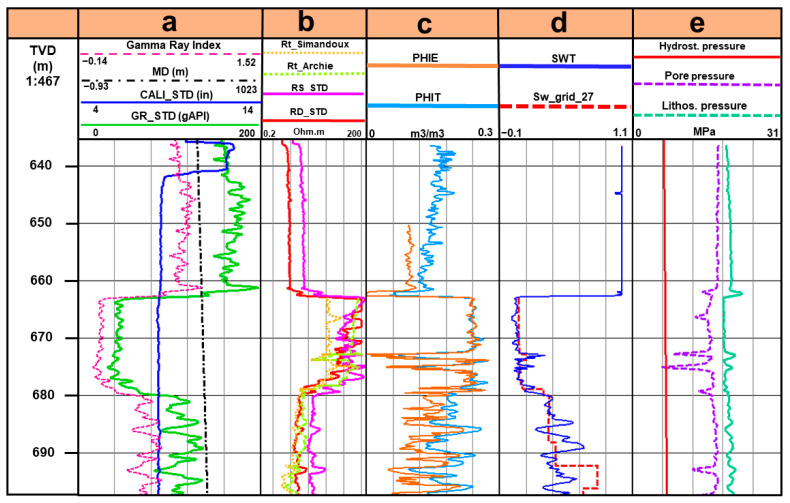
The section displays different well logs of the central well 7324/8-1. The well section is divided into five tracks (**a**–**e**), separated by black vertical lines. TVD on the far left stands for true vertical depth. (**a**) Gamma-ray index log, measured depth (MD), caliper log (CALI_STD) and gamma-ray log (GR_STD). (**b**) Resistivity log modeled using the Simandoux equation, another resistivity log modeled using Archie’s law, shallow and deep resistivity logs, respectively (RS_STD and RD_STD). (**c**) Effective (PHIE) and total porosity (PHIT) logs, respectively. (**d**) Water saturation log (SWT) and the other one (Sw_grid_27) is for water saturation extracted from a simulation grid at the start of production in the year 2027. (**e**) Pressure logs, including hydrostatic, pore and lithostatic pressures.

Different well logs (Figure 4) from the central well (7324/8-1) were analyzed together with the inverted CSEM results to construct and calibrate the rock physics model of Equation (2) for realistic resistivity models. The well logs show different property information about the reservoir with depth. For instance, the gamma-ray log (green curve) is used for the gamma-ray index log (pink curve) (Figure 4a) which is further used to study the clay content across the reservoir, and whether the reservoir can be considered as clean. There is a clear drop in both log readings at approximately 660 m depth and down to 680 m, demarcating the main reservoir zone with most likely little clay content. Moreover, Figure 4b shows how the resistivity readings are displaying anomalously high values related to hydrocarbons in the pore space. Well log measurements can have huge fluctuations as the well penetrates through different lithologies with different matrix compositions and pore space fluid content with different saturations. For instance, we can see how the resistivity readings in Figure 4b can reach values beyond 200 ohm-m (beyond the saturation point of the logging tool) at the most resistive parts of the oil reservoir. When investigating hydrocarbon reservoirs, one important property is porosity. In Figure 4c, two different porosity logs are presented, effective porosity (orange curve) and total porosity (blue curve). The main difference between the two is that total porosity looks at total porosity including clay bound water (CBW), while effective porosity excludes CBW. If the formation is clay free, then total porosity is equal to effective porosity, and this is what we see within the reservoir in Figure 4c. Furthermore, we can see that the porosity values are higher across the reservoir zone, indicating the potential high quality of the reservoir. Figure 4d shows water saturation logs. The first one, total water saturation (Swt), in blue, is a continuous log reading, while the other one in red is extracted from a dynamic simulation grid of the water saturation in the year 2027, just before the reservoir starts producing as assumed. Since each saturation value is extracted from a specific cell at different depths, this log looks like a staircase. Figure 4d clearly shows that water saturation (both logs) drops across the reservoir zone (660–680 m). Figure 4e presents three different pressure logs, hydrostatic pressure (red curve), pore pressure (violet) and lithostatic pressure (green). The pore pressure log is modeled from the other pressure logs to study the effect of pore pressure on the modeled resistivity logs (Archie and Simandoux, in Figure 4b), and the main observation here is that most likely there is no overpressure in the reservoir. 

Therefore, more sophisticated petrophysical models, such as the shaly sand model described by the Simandoux equation [37], are not selected for the inclusion of clays. When using shaly sand models, any considerable amounts of clay will most likely reduce the bulk resistivity of the reservoir rock. The clay modeling is calibrated with well 7324/8-1, proving that clay incorporation does not result in the observed high CSEM anomalies (orange dotted curve in Figure 4b). This indicates that the reservoir rock at Wisting has a negligible influence from clays. Moreover, Meunier [38] and Alvarez et al. [39] characterize the two main formations of the reservoir—Stø and Nordmela—as consisting of well-sorted quartz-rich sandstone with negligible clay content, providing further evidence for the clay-free assumption we applied in Equation (2). 

**Figure 5:**
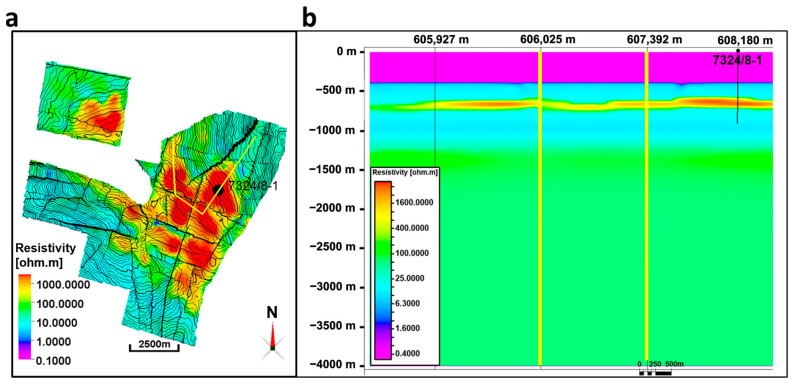
Three-dimensional inversion results from real CSEM data acquired in 2014. (**a**) Resistivity map across top Stø horizon. The thick yellow line is an intersection line going through the most pronounced resistivity anomalies and the thin wiggly black lines throughout the map are contour lines. (**b**) Resistivity cross section along the thick yellow line shown in subfigure (**a**). The two yellow vertical lines on this resistivity section indicate the two corners on the intersection line of subfigure (**a**).

The inverted vertical resistivity map from the CSEM data acquired over the Wisting field in 2014 highlights several pronounced resistive anomalies (Figure 5a). The resistive anomaly in the cross-section (Figure 5b) at the location of well 7324/8-1 corresponds to the resistive anomaly in the logging results (Figure 4b). This enables us to use log information together with inversion results to select reliable petrophysical parameters. Our approach uses the deep resistivity log reading as a starting point for resistivity model construction and calibration. Water saturation is obtained from the well log, while brine resistivity is set equal to 0.18 ohm-m, based on previous research [28]. It is, however, worth mentioning that CSEM and well-log resistivities look at two different scales of the same physical properties, especially when resistivity anisotropy is present [28]. 

**Figure 6:**
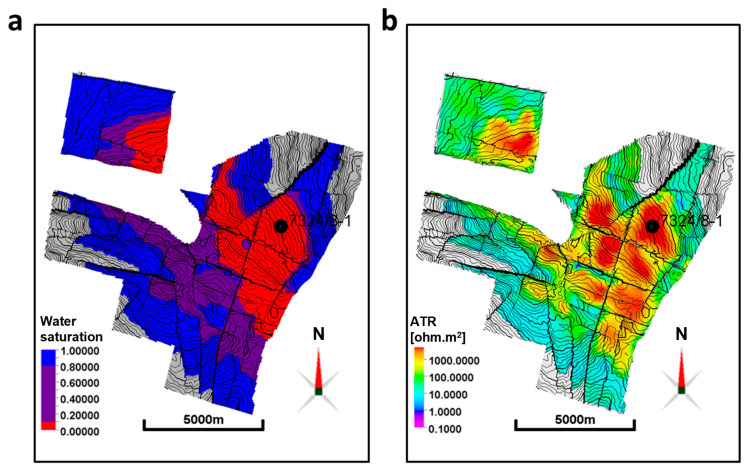
Maps used in our wettability analysis. (**a**) Water saturation map highlighting in red the distribution of water saturation cells equal to or below the connate water saturation threshold, being considered oil-wet and receiving larger saturation exponents in our modeling. The indigo color displays water saturation cells above the threshold value and below 0.8 (cells that contain a mixture of brine and oil). (**b**) ATR map from CSEM inversion for comparison. It is clear from subfigure (**b**) that the east flank is responsible for the most pronounced anomalies, and that the water saturation values in subfigure (**a**) will account for these high anomalies as they will obtain larger saturation exponents and hence result in higher resistivities. The thin wiggly black lines throughout the maps are contour lines. The grey areas are regions without values of the displayed properties.

The saturation exponent influenced by wettability [40] should be carefully selected in resistivity model construction for the CSEM response modeling. The model developed here is calibrated with well 7324/8-1 and elaborates on wettability in conjunction with the conclusions made by Mungan and Moore [36] on oil-wet formations. The model deals with the non-linearity of the saturation exponent in oil-wet formations, implying a dynamic water saturation. The saturation exponent should increase significantly in oil-wet formations to account for the isolated water globules in the larger pores, which will not be able to conduct current [41]. Moreover, the wettability effects become more prominent as the brine saturation decreases. This establishes a connate water saturation (Sw, connate) threshold or cut-off below which the rock changes its wettability from a state of mixed wettability of brine and oil to becoming oil-wet, resulting in a larger saturation exponent. Alvarez et al. [39] use a saturation exponent equal to 2.8 in their Wisting resistivity modeling. By incorporating information from both capillary pressure curves and free water level (FWL) depth maps, the connate water saturation threshold is set to 10%. Figure 6 summarizes the influences that such a cut-off value will have on the distribution of resistivity anomalies. The red-colored areas of the water saturation map (Figure 6a) are considered oil-wet, receiving larger saturation exponents. Hence, they will most likely show the highest resistivities, since a larger saturation exponent produces larger resistivities, assuming that all other parameters are kept constant.

The CSEM inversion results are used in our study to calibrate the developed rock physics model [29]. A comparison between the ATR map extracted from the CSEM inversion and the water saturation map highlighting the implications of the suggested rock physics framework is shown in Figure 6. From this, we can adjust the different parameters of the rock physics framework to obtain the smallest misfit between the two resistivity models. A good accordance is observed between the red-colored area of the water saturation map in Figure 6a and the high anomalies of the ATR map recovered from CSEM inversion in Figure 6b. 

**Figure 7:**
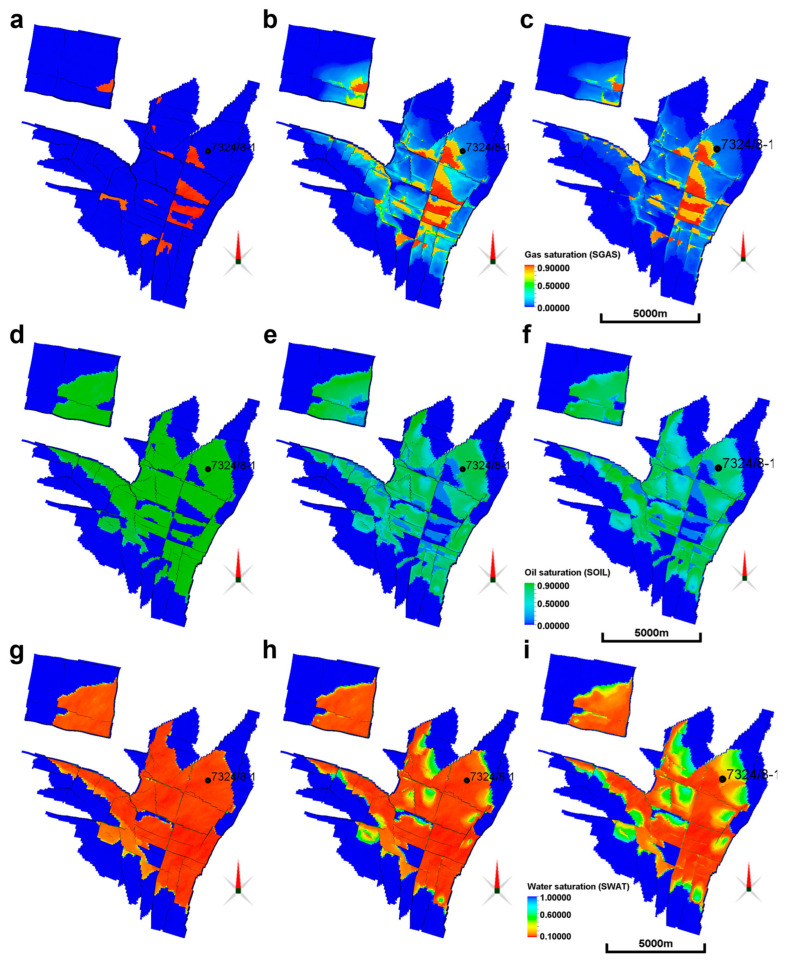
Dynamic saturation maps for the base case and two monitoring cases (years 2027, 2032 and 2042). Top row: gas saturation maps pertaining to (**a**) base case in 2027, (**b**) monitoring case in 2032 and (**c**) monitoring case in 2042. Middle row: oil saturation maps pertaining to (**d**) base case in 2027, (**e**) monitoring case in 2032 and (**f**) monitoring case in 2042. Bottom row: water saturation maps pertaining to (**g**) base case in 2027, (**h**) monitoring case in 2032 and (**i**) monitoring case in 2042. The color bar pertaining to each row is shown on the last figure of each row. The well 7324/8-1 is highlighted by a black dot on each figure.

Figure 7 presents dynamic maps for three different saturations, gas saturation (Figure 7a–c), oil saturation (Figure 7d–f) and water saturation (Figure 7g–i) at different production phases. In the water saturation maps, both oil and gas production effects are merged. The different saturation maps of Figure 7 give an idea of how the oil production at Wisting is simulated as a function of time. The saturation fractions should always sum up to 1 when different fluids are present in the pore space, according to $S_{w}+S_{o}+S_{g}=1$, where S stands for saturation and the subscripts W, O and G for water, oil and gas, respectively. Thus, in our case, the water saturation is calculated as $S_{w}=1-\left(S_{o}+S_{g}\right)$. Figure 7g–i shows how the water saturation increases with time as a result of oil production while produced oil is replaced by water and gas. At the beginning of production, the gas caps scattered over the field, as shown in Figure 7a, call for further refinement of our resistivity model. These gas caps deserve special treatment in terms of the saturation exponent *n*. The latter should most likely be less than 2 in gas-bearing formations, introducing the necessity for choosing another threshold value for the gas saturation which, for the reader’s convenience, is given the name critical gas saturation. This may consequently demarcate the transition from oil-wet rocks with low water saturation combined with a large saturation exponent on one hand, and gas-bearing zones with relatively high gas saturation combined with a lower saturation exponent on the other hand. After multiple attempts, a gas saturation threshold value equal to 70% is deemed to produce the desired models. In the desired models, the generated resistivity should be consistent with our understanding of the spatial distribution and magnitude of the most resistive targets, and this should also conform with the inverted 3D CSEM resistivity maps [29].

### 3.3. Constructed Resistivity Models

From the thorough study in the previous subsection, a non-linear saturation exponent is applied, having its maximum value of 2.5 at the top of the reservoir and non-linearly decreasing with depth following the overall water saturation trend. Note that this only applies for intervals where the water saturation is lower than the connate water saturation of 10%. On the other hand, if the gas saturation is higher than the critical gas saturation, the saturation exponent *n* is assigned the value 1.8. To summarize, there are two main factors that decide what kind of value the saturation exponent is assigned, and these are the connate water saturation and critical gas saturation thresholds. Table 1 summarizes the subsurface parameters used to construct and calibrate the resulting resistivity distribution. The presented production strategy spans the years 2027 to 2057, implying that the oil saturation will non-linearly decrease with time as depicted by the saturation maps of Figure 7. This non-linear decrease in the oil saturation with time is inferred from the observed exponential decay in the oil production rates. By using Archie’s law, water saturation simulation models representing a specific production strategy through time are transformed into corresponding resistivity models. This provides us with the possibility of digging into the 4D production-induced effects through the synthetic modeling of time-lapse CSEM data, which will be the subject of the next section.

**Figure 8:**
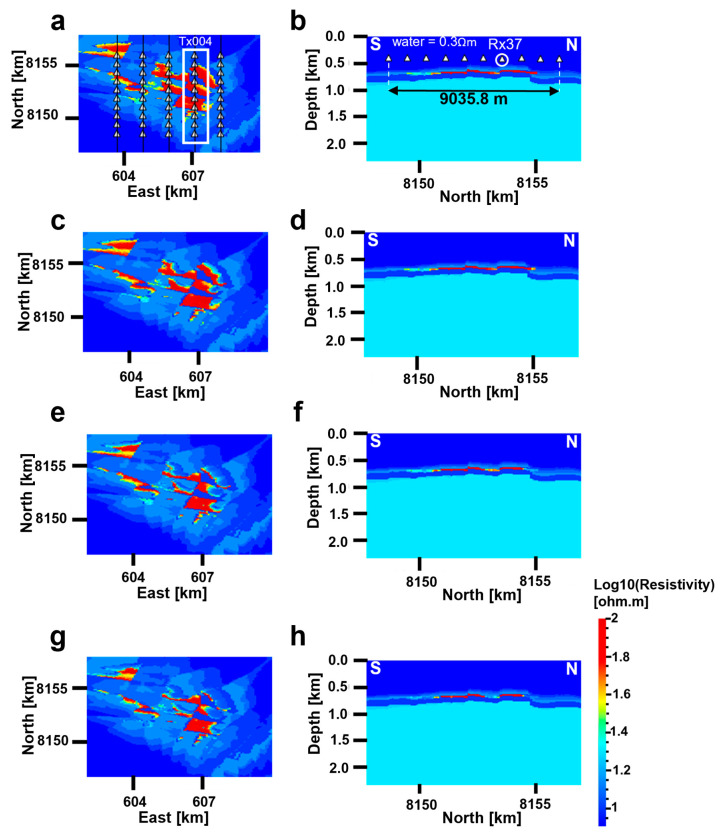
Resistivity models at different time steps. (**a**) Horizontal model section at 660 m depth in 2027, (**b**) vertical model section along Tx004 in 2027. (**c**) Horizontal model section at 660 m depth in 2032, (**d**) vertical model section along Tx004 in 2032. (**e**) Horizontal model section at 660 m depth in 2042, (**f**) vertical model section along Tx004 in 2042. (**g**) Horizontal model section at 660 m depth in 2057, (**h**) vertical model section along Tx004 in 2057. The white triangles are receivers and the black vertical lines going through them are towlines. The annotation Tx004 is the name given to the fourth towline and Rx37 is the name of a receiver. The colorbar is logarithmically scaled.

The rock physics model with the highlighted parameters in Table 1 is used to transform the detailed water saturation simulations to resistivity distributions, as shown in Figure 8. Note that resistivity changes due to fluid substitution are only introduced within the reservoir interval, while the background resistivity is fixed. Resistivity models are then generated every five years from 2027 to 2057, assuming production lasts for 30 years. 

## 4. Time-Lapse CSEM Data Modeling

### 4.1. Acquisition Settings

Figure 8 shows horizontal and vertical model sections for the years 2027 (Figure 8a,b), 2032 (Figure 8c,d), 2042 (Figure 8e,f) and 2057 (Figure 8g,h), respectively. The acquisition setting is also shown together with the models in Figure 8a,b. The resistivity cross sections are along the fourth towline Tx004 as indicated by the white rectangle in Figure 8a. The resistivity models represent the base and three monitoring cases, respectively. Since warm color represents the resistivity of the reservoir, one can observe that after 30 years of hydrocarbon production (from 2027 to 2057), most of the resistive fluid is replaced by water and what remains is a few resistive patches scattered all over the modeled region. Therefore, it is obvious how the resistivity decreases across the field due to oil production. Projected on top of Figure 8a is a synthetic CSEM survey array as indicated by white triangles for the receivers and black vertical lines for the towlines. The synthetic survey consists of 10 seabed receivers along each towline, with 5 parallel towlines that gives a total of 50 seabed receivers. Receiver inline and crossline spacings are 1 km and 1.5 km, respectively. The towline spacing is accordingly 1.5 km as well. Note that the water depth is 400 m, and the total model depth is 9000 m even if the resistivity cross sections only show depths to 2000 m. The model depth is found based on the following rule: total model depth = max water depth + 3.3 × skin depth. The skin depth, δ, is defined as [42]:

$$\delta =500\sqrt{\frac{\rho }{f}},\;$$

where *ρ* and *f* represent resistivity and frequency, respectively. 

The highlighted receiver Rx37 in Figure 8b is the receiver from which the results of the next section stem. The reason behind our choice of this receiver is the fact that it is perfectly situated between two different producing segments of a fault, as shown in the resistivity cross sections (Figure 8b–h), and where we see that in the year 2057, most of the southern segment of the fault proximal and relative to this receiver shows low resistivity. This helps us to understand how this production-induced resistivity change is reflected in the synthetically modeled data, and how well we can capture such effects in the data domain. 

The resistivity models presented in Figure 8 are used as inputs in an FDTD workflow, SBLwiz developed by EMGS [31], to generate synthetic CSEM data at eight different frequencies: 7.5, 9.0, 12.0, 13.0, 16.0, 17.0, 25.0 and 30.0 Hz. The mesh used in the forward modeling is a uniform grid composed of 583 × 566 × 900 cells. Each cell is 10 m in the vertical direction and 100 m in the horizontal directions to capture the detailed changes during production. The model’s spatial coverage is 58.3 × 56.6 × 9 km. The horizontal components of both the electric E and the magnetic H field are simulated. The source dipole used in the modeling is 278 m long, the source current is 1250 A and the source is kept 30 m above the seabed. The computational runtime required for each 3D modeling timestep varies from 10 to 19 h. Differences between the base and monitoring input resistivity models are reflected in the modeled CSEM data. We also carried out comparisons with layering models between a 1D analytical solution and the 3D approximation solution to verify the correctness of the software. Since the relevant results have been published [31,43,44,45], the comparison is not shown here. 

### 4.2. CSEM Data Modeling and Time-Lapse Production-Induced Responses

**Figure 9:**
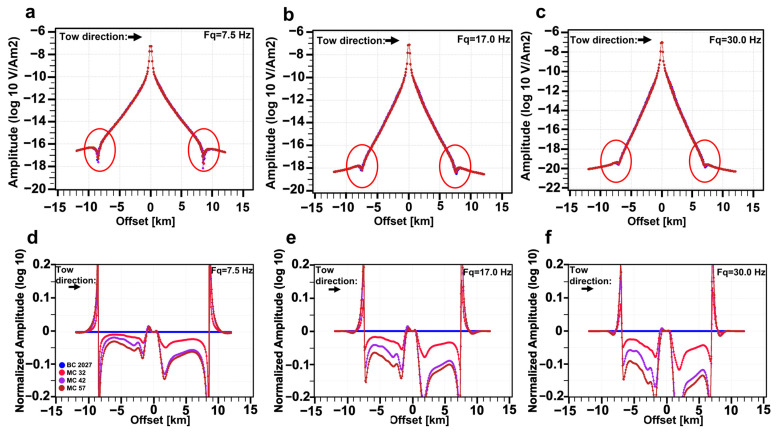
Three-dimensional CSEM-modeling responses for the base case (BC 27) and three monitoring cases (MC 32, MC 42 and MC 57). Top row: the inline electric field E_x_ according to the transmitter trajectory shown by the towline Tx004 and recorded at the receiver Rx37, as highlighted by Figure 8, at frequencies (**a**) 7.5Hz, (**b**) 17.0 Hz and (**c**) 30.0Hz. Bottom row: normalized amplitude of the inline electric field E_x_ according to the transmitter trajectory shown by the towline Tx004 and recorded at the receiver Rx37 as highlighted by Figure 8 at frequencies (**d**) 7.5Hz, (**e**) 17.0 Hz and (**f**) 30.0 Hz. One legend applies to all plots and is located on the plot in the lower left corner. The *y*-axis is logarithmically scaled. The tow direction is indicated with an arrow on the upper left corner, and frequency on the upper right corner of each plot. The red circles on the subfigures of the first row highlight a spiky feature that is observed at relatively large offsets, when the amplitude of the electric fields is below the noise floor.

The predicted onset of production is in the base year 2027. Figure 9a–c shows the amplitude curves of the horizontal inline E_x_ field at different frequencies for different timesteps. Figure 9d–f shows the normalized amplitude (NA) curves for the same electric fields. The NA is calculated as the ratio of the amplitudes of the electric fields from the base case in the denominator and the amplitudes of the electric fields from the monitoring case of each timestep in the numerator. Important time-lapse information can also be extracted from the phase of the electromagnetic fields, but this is not considered here. Figure 9 shows how higher frequencies lead to more pronounced 4D effects in the NA curves. For instance, we obtain a greater separation between the NA curves for 17.0 Hz than for 7.5 Hz (Figure 9d,e). Deviating NA curves from the blue curve, representing the base case, implies that the electric field amplitudes from the considered monitoring case have changed, and this change is already strong in 2032, after five years of production. Later monitoring cases result in a larger NA variation (larger resistivity differences between the base case and the monitoring case), and this can be traced back to ongoing oil production and hence decreasing resistivities with time in the reservoir. The red circles in Figure 9a–c highlight a spiky feature that we can observe at relatively large offsets and when the amplitude of the electric fields is below the noise floor. Most likely, this spiky feature encompasses the airwave effect [46]. In our case, the water depth is 400 m, and the airwave effect is larger in shallow water (<1.5 km). What is interesting to observe here in terms of time-lapse CSEM is how the separation between the curves increases with increasing frequency and how the out-tow direction (away from the receiver Rx37) leads to more pronounced separation than the in-tow direction (towards the receiver Rx37). This can be explained using the resistivity cross sections of Figure 8b,d,f and h, where we can see that on the right of the receiver Rx37, there is a drop in resistivity between the base case in the year 2027 and the later monitoring cases at approximately 1500 m from the receiver towards north. This resistivity drop is reflected in our data in Figure 9d–f by the clear separation in the NA curves of the out-tow direction starting at about 1500 m offset. CSEM-modeled data can capture 4D production-induced changes in the reservoir. Furthermore, higher frequencies, such as 30 Hz, give better results in terms of resolving these 4D effects compared to the modeled low frequency, such as 7.5 Hz. However, high-frequency signals attenuate faster than relatively low-frequency signals. Therefore, a combination of relatively high background resistivity and shallow burial depth might have contributed to a setting that is favorable for these high frequencies.

**Figure 10:**
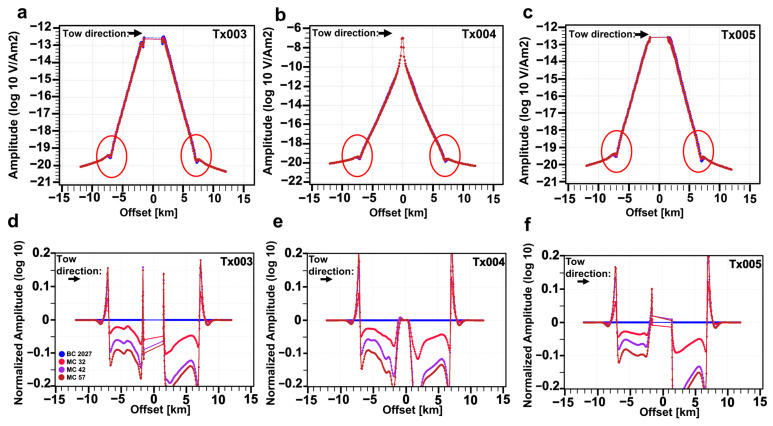
Three-dimensional CSEM-modeling responses for the base case (BC 2027) and three monitoring cases (MC 32, MC 42 and MC 57). Top row: the inline electric field E_x_ at 30 Hz frequency and recorded at the receiver Rx37, as highlighted by Figure 8, according to the transmitter trajectory shown by towline (**a**) Tx003, (**b**) Tx004 and (**c**) Tx005. Bottom row: Normalized amplitude of the inline electric field E_x_ at 30 Hz frequency and recorded at the receiver Rx37 as highlighted by Figure 8 according to the transmitter trajectory shown by towline (**d**) Tx003, (**e**) Tx004 and (**f**) Tx005. One legend applies to all plots and is located on the plot in the lower left corner. The *y*-axis is logarithmically scaled. The tow direction is indicated with an arrow on the upper left corner, and towline on the upper right corner of each plot. The red circles on the subfigures of the first row highlight a spiky feature that is observed at relatively large offsets, when the amplitude of the electric fields is below the noise floor.

Another way of studying the results is to consider the different towlines of the survey and to investigate whether other towlines provide more information about the depleting reservoir. Figure 10 shows the responses acquired along different towlines at 30 Hz. Towlines Tx003 and Tx005 are, respectively, the ones on the left and right of the framed towline in Figure 8a. Figure 10a,d shows the results of Tx003 and Figure 10c,f shows results for Tx005 (these are side lines). The red circles in Figure 10a–c again highlight the airwave effects and noises in the simulated data. Figure 10 demonstrates that different towlines show the same pattern in the 4D effects highlighted in our data, emphasizing the greater separation between the NA curves in the out-tow direction. The gap that we see in the data for towlines Tx003 and Tx005 around zero offset is because the receiver is not inline but azimuthal with respect to the towline, i.e., the electric dipole or the transmitter is not going along the receivers like towline Tx004 (Figure 10b). 

### 4.3. Attribute Analysis of the Modeled CSEM Responses 

**Figure 11:**
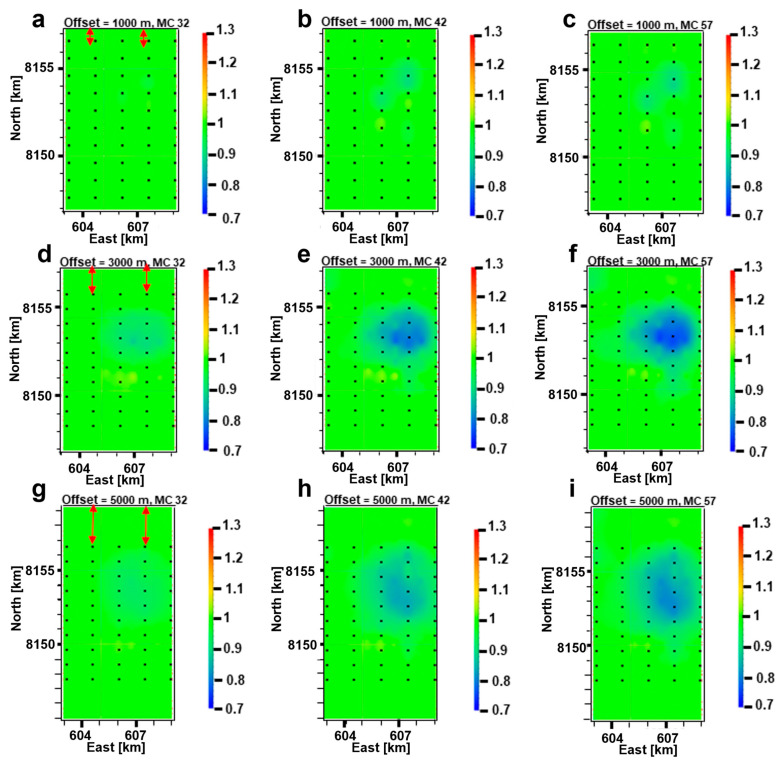
Normalized amplitude (NA) 2D maps of the E_x_-field component for three monitoring cases (years 2032, 2042 and 2057) and three different source–receiver offsets (1000, 3000 and 5000 m). Top row: 2D NA maps at 1000 m source–receiver offset pertaining to a specific monitoring case (**a**) 2032, (**b**) 2042 and (**c**) 2057. Middle row: 2D NA maps at 3000 m source–receiver offset pertaining to a specific monitoring case (**d**) 2032, (**e**) 2042 and (**f**) 2057. Bottom row: 2D NA maps at 5000 m source–receiver offset pertaining to a specific monitoring case (**g**) 2032, (**h**) 2042 and (**i**) 2057. The title on top of each plot highlights the considered offset and monitoring case (MC), where years 2032, 2042 and 2057 are referred to as 32, 42 and 57, respectively. The frequency considered for all plots is 7.5 Hz. The black dots are receiver positions. The red arrows on the subfigures of the first column point out how data coverage beyond the first and last receivers on each towline increases along with increasing offset.

Figure 11 presents 2D maps constructed from the NA curves. Figure 11 depicts the 2D spatial interpolation of each NA data pair stemming from each receiver of the survey. Each receiver gives two electric field amplitude readings for a specific offset, one for the in-tow direction (towards the receiver) and one for the out-tow direction (away from the receiver). These pairs of data are normalized as described earlier and populated all over this 2D map. Then an interpolation approach assures that these pairs of data points are evenly smoothed out, covering the whole survey area. We assume that each receiver gives one common middle point (CMP) for the in-tow direction and another one for the out-tow direction. Therefore, the distance between each data point and the transmitter is half the offset, and in our case an offset of 3000 m implies 1500 m between the transmitter and the data point in each direction, giving two data points at these locations. Using the red arrows in Figure 11a,d,g, we point out how data coverage beyond the first and last receivers on each towline increases with increasing offset. These plots might be misleading if penetration depth varied dramatically within the reservoir. However, in our case, useful information can be extracted from these 2D maps due to sufficient penetration depth. Figure 11 presents three different source–receiver offsets 1000 (Figure 11a–c), 3000 (Figure 11d–f) and 5000 m (Figure 11g–i). Each row in the figure presents three different monitoring cases in 2032, 2042, and 2057. The frequency is 7.5 Hz, which is the lowest frequency modeled. We selected it due to the considerations of the penetration depth of the signal and the data sensitivity to a target. 

Figure 11 depicts discernible production-induced changes represented by the blue anomalies. A producing hydrocarbon field is expected to have decreasing petroleum volumes with time and hence lower resistivity values due to water injection. Towards the end of the lifetime of a producing field, most of the target resistor will be gone and the subsurface model is without the target resistor embedded in it. Figure 11 underpins this fact with the anomalies being lower than unity, where the normalized amplitude has been defined earlier in the paper. The prevailing light-green color is for unity and implies that there is no difference between the data of the base case and the monitoring case. This further implies that most likely no significant change in subsurface resistivity has occurred. On the other hand, the blue color suggests that the subsurface resistivity has changed between the two modeled surveys, and hence 4D effects are reflected in our data. Studying different offsets can best capture these 4D effects. One can observe that it is the anomalies at 3000 m offset (Figure 11d–f) that reflect these resistivity changes the best. This is reasonable in terms of the investigation depth of the data and the depth of the reservoir (662 m). A commonly suggested approximation in the CSEM community is that the response at 1500 distance is most sensitive to subsurface around 750 m deep, i.e., half the distance. This explains why Figure 11d–f best captures the 4D effects. Interestingly, the time-lapse effects are present in each time-step and already detectable after five years of production in the year 2032 when the offset is 3000 m. It is also clear that the EM anomaly becomes more significant over time while hydrocarbon production takes place. A clear and good spatial match exists between these anomalies and the resistivity maps in Figure 8a,b, as we can see that the epicenter of the anomaly is where most of the production is predicted to take place. Clearly, the CSEM data can capture production-induced changes in the reservoir resistivity at the time step of five years. Due to the relatively low cost of a CSEM survey, it is a cost-effective monitoring tool for oil production progress and safety. 

**Figure 12:**
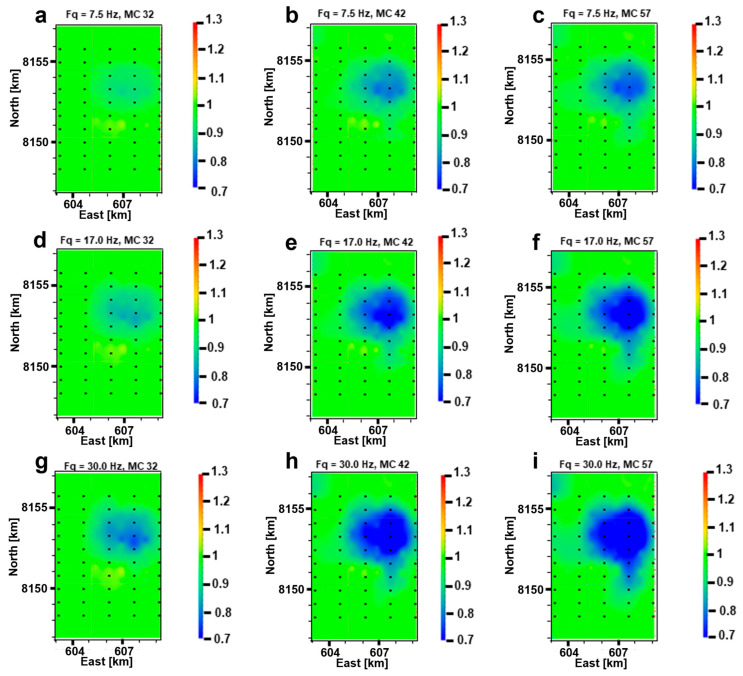
Normalized amplitude (NA) 2D maps of the E_x_-field component for three monitoring cases (years 2032, 2042 and 2057) and three different source frequencies (7.5, 17.0 and 30.0 Hz). Top row: 2D NA maps at 7.5 Hz m frequency pertaining to a specific monitoring case (**a**) 2032, (**b**) 2042 and (**c**) 2057. Middle row: 2D NA maps at 17.0 Hz frequency pertaining to a specific monitoring case (**d**) 2032, (**e**) 2042 and (**f**) 2057. Bottom row: 2D NA maps at 30.0 Hz frequency pertaining to a specific monitoring case (**g**) 2032, (**h**) 2042 and (**i**) 2057. The title on top of each plot highlights the considered frequency (Fq) and monitoring case (MC), where years 2032, 2042 and 2057 are referred to as 32, 42 and 57, respectively. The source–receiver offset considered for all plots is 3000 m. The black dots are receiver positions.

Since the 3000 m offset best reflects the subsurface resistivity changes during production, we then studied which frequency provides the strongest signals during oil production. Figure 12 shows the CSEM responses for the different monitoring cases at different frequencies, while the offset is fixed to 3000 m. The results indicate that more pronounced anomalies evolve with time between the different monitoring cases and that the highest frequency (30 Hz) best accounts for the bulk of subsurface resistivity changes. Note that already, after five years of production in the year 2032, 30 Hz frequency data can resolve distinct resistivity alterations compared to the base case. Even though a 30 Hz transmitting frequency is very high in a marine environment, it is feasible to use it nowadays with a Scripps instrument and an EMGS instrument.

**Figure 13:**
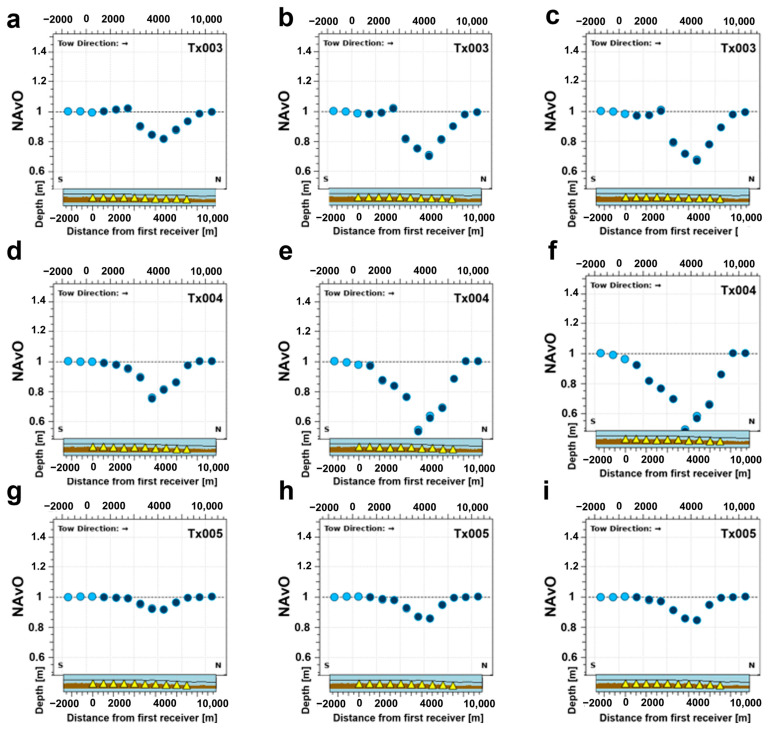
Normalized amplitude (NA) line summary plots of the E_x_-field inline response at 3000 m offset for three monitoring cases (year 2032, 2042 and 2057) and three different towlines (Tx003, Tx004 and Tx005). Top row: NA line summary plots for the towline Tx003 pertaining to a specific monitoring case (**a**) 2032; (**b**) 2042 and (**c**) 2057. Middle row: NA line summary plots for the towline Tx004 pertaining to a specific monitoring case (**d**) 2032; (**e**) 2042 and (**f**) 2057. Bottom row: NA line summary plots for the towline Tx005 pertaining to a specific monitoring case (**g**) 2032; (**h**) 2042 and (**i**) 2057. The considered towline is indicated on the top right corner of each plot. The source–receiver offset is 3000 m, and the frequency is 30.0 Hz for all plots. The light blue color is for the in-tow response and the darker blue color is for the out-tow response. The small section below each plot shows a cross-section along the considered towline including the transmitter trajectory with depth below sea surface (black line), receivers (yellow triangles) and the receiver positions on the sea bottom (brown). The *x*-axis is for the distance from the first receiver in the considered towline and the tow direction is indicated with an arrow on the upper left corner of each plot.

A combination of a 3000 m transmitter–receiver offset and transmitting at 30 Hz gives the most promising results in terms of detecting resistivity alterations in a producing oil reservoir using the CSEM method at Wisting. Figure 13 presents line summary plots along each of the following towlines: Tx003, Tx004 and Tx005 and for each monitoring case of the three discussed throughout this paper (2032, 2042 and 2057). In Figure 13, each subfigure presents data points from all receivers that are positioned along the considered towline. This means that we should expect at least 10 different CMP datapoints since we have 10 receivers along each towline. Bearing in mind that each receiver gives two data points, one for the in-tow and one for the out-tow directions, we should then expect the data points to be twice the number of receivers in Figure 13a–i. Each of the 20 data points will be located at the middle of the offset, i.e., a distance between the transmitter and the receiver that corresponds to half the offset. It might be that the subfigures display fewer than 20 data points, simply because in the middle part, the in-tow and out-tow data points are possibly overlapping. The *x*-axis here is not the offset but the distance to the first receiver on the considered towline. Figure 13 proves that towline Tx004 is the one that best represents the production-induced resistivity changes in the reservoir, because it is along this towline that we observe most of the oil production in the time-lapse resistivity models (Figure 8). It is most likely that the injection flow and transmissibility patterns across the reservoir play a role in resistivity changes at different time steps. We believe that a reasonable extension of this study is to invert the modeled EM data to 3D resistivity distributions and investigate the repeatability requirements in the context of time lapse.

One recent research paper claims that the repeatability requirements for acquisition parameters in time-lapse studies can be relaxed when inverted resistivity volumes are considered [13], i.e., when results are examined in the model domain rather than the data domain. Another point worth emphasizing is the possibility of extending the presented workflow to other CSEM applications such as CO_2_-storage monitoring.

### 4.4. ATR Maps of Resistivity Models

Dynamic reservoir simulation scenarios for oil production were converted to representative resistivity distributions (Figure 8) through geologically consistent rock physics models. These spatial resistivity distributions may not only be used as inputs in a FDTD workflow to generate synthetic CSEM data, but also be transformed into ATR maps. ATR is a good way of digging out information from real field data, because ATR combines the resistor resistivity and geometry. Therefore, the developed resistivity distributions can be used to: (1) generate the CSEM synthetic responses and (2) to make the ATR maps, assuming the constructed resistivity models come from inversions.

**Figure 14:**
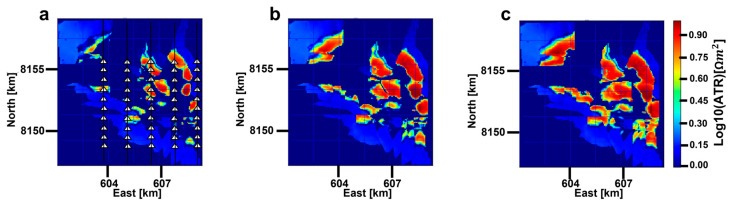
Relative difference ATR maps, with the base case in 2027 as reference for (**a**) monitoring case in 2032, (**b**) monitoring case in year 2042 and (**c**) monitoring case in year 2057. The white triangles are receivers and the black vertical lines going through them are towlines. The colorbar is logarithmically scaled.

As a parameter in the model domain, we can use ATR maps (Figure 14) to investigate our results for CSEM time-lapse feasibility in the model domain. Figure 14 presents three relative difference (RD) ATR maps for three monitoring cases in 2032, 2042 and 2057, with the base case ATR map for the year 2027 as a reference. The ATR maps have been constructed using Equation (1), where the difference between resistor and background resistivities have been summed up across the reservoir thickness. The RD ATR was calculated as the absolute value of the difference between the base case and monitoring case ATR values divided by the base case ATR. Figure 14a shows that the RD ATR after five years of production (2027 to 2032) is pronounced, and after 10 years of production (year 42), in Figure 14b, the RD is even more prominent. As for the year 2057, towards the end of production, the resistivity change is less prominent compared to the year 2042 since most of the oil has been produced by that time. Clearly, the production-induced changes in the pore fluid of the producing hydrocarbon field are captured. Figure 14 is a simple extension of our subsurface resistivity distribution in Figure 8, but it still provides us with valuable insight when this figure is compared with Figure 11 and Figure 12. Both CSEM responses and ATR maps confirm the feasibility of time-lapse CSEM for oil production monitoring at Wisting, by capturing time-lapse anomalies. They depict the same pattern, relative to time, where the anomalies become larger further in time. However, there are obvious differences between the two approaches. One of the differences is resolution, where Figure 11 and Figure 12 display the time-lapse anomaly as a smoothed volume. We do not see in Figure 12, for example, the tiny spots and patches we see in Figure 14. Another difference between the two approaches is that the production taking place in the northwest of the survey is weakly reflected in the data domain (Figure 12h) in contrast to the ATR map (Figure 14b). This indicates that the CSEM responses only provide a general picture of the production-induced reservoir change and ATR maps can provide a more detailed picture of it with the available models. However, in order to convert the ATR maps, inversion should be carried out beforehand. This leads to longer planning time due to the heavy computation cost for 3D marine CSEM inversion. 

## 5. Discussion

A rock physics model was used to convert the dynamic water saturation simulations of Wisting to resistivity distributions for each time step at different production phases. These resistivity distributions were further used as inputs in an FDTD software (SBLwiz version v3.0.4.24765) to model CSEM data and for ATR map calculation. Both synthetic CSEM data and ATR maps were analyzed for CSEM time-lapse feasibility for oil production monitoring. We called them Approach 1 and Approach 2 for convenience purposes. Approach 1 is in the data domain and Approach 2 is in the model domain.

Both approaches are based on our constructed resistivity models. Therefore, resistivity model construction is crucial in our study. A modified version of Archie’s law was applied for the resistivity model construction, assuming a non-linear saturation exponent. 

This non-linear saturation exponent accounts for the anomalously high resistivities when the formation is oil-wet. Especially, the reservoir being oil-wet at Wisting has been proposed in several studies [29,39,47]. By establishing a connate water saturation threshold, we can increase the saturation exponent when the formation water saturation is below this threshold. Then the model is calibrated with the deep resistivity log reading from well 7324/8-1. Furthermore, the gas present across the field is accounted for by introducing another threshold called the critical gas saturation, below which the saturation exponent is less than 2. Including the clay effect in our modeling did not result in the observed high resistivity anomalies, and hence it was discarded. The final base model is consistent with the inverted resistivity distributions shared by our industrial partner (Figure 6), validating our assumptions regarding the reservoir being oil-wet when the reservoir water saturation is below connate water saturation and that the clay effect is not prominent. This implies that the applied Archie’s law is sufficient, without the need to use other available shaly sand approximations. One should note that 7324/8-1 is the only well used for our rock physics model construction and calibration. The inclusion of other wells may further improve the model construction. However, this well matches the CSEM results very well, and it is representative and hence was selected. Another issue is that CSEM data and well logs are sensitive to resistivities at different scales, especially when resistivity anisotropy is present. Even though anisotropy is not reported at Wisting, one should note that the scale difference between well log data and CSEM data is not considered for the resistivity model construction in our study. In general, our way of constructing resistivity models for Wisting is realistic and reliable for the presented CSEM time-lapse study. 

With Approach 1, the synthetic CSEM data are simulated using an FDTD scheme. The modeled fields were used to construct 2D maps of the NA curves (Figure 11 and Figure 12). By analyzing the 2D NA maps, we could study how the reservoir resistivity changes during production. The simulated data highlight production-induced effects to varying extents for the whole frequency band. Not surprisingly, more pronounced responses are obtained for higher frequencies. A combination of shallow burial depth and high background resistivity contributed to this, otherwise, limited skin depth would not allow the high-frequency signals to penetrate the entire reservoir. In our case, a frequency and offset equal to 30 Hz and 3000 m, respectively, gave the best sensitivity to 4D production effects, and the CSEM data detected changes in subsurface resistivity in all monitoring surveys (Figure 11 and Figure 12). The inline receivers of the fourth towline Tx004 in our survey are deemed to give the best results in terms of capturing the production-induced alterations in subsurface resistivity due to the most influenced regions by production being below these receivers. 

With Approach 2, ATR maps (Figure 14) provide another way of looking at our modeled resistivity distributions. By combining the ATR maps (Figure 14) with the NA maps constructed from synthetic CSEM responses (Figure 11 and Figure 12), the feasibility of the presented workflow in proving production-induced changes in the fluid resistivities is well demonstrated. This suggests that time-lapse CSEM for oil production monitoring at different production phases might be applied together with time-lapse seismic or other methods, ensuring a broader understanding of the field development during production. 

Both approaches have advantages and disadvantages. The advantage of Approach 1 is that we can use the electric and magnetic fields to monitor production without involving heavy computation. However, this approach may miss some detailed features that might occur during production. This is because, when we study these CSEM responses in the context of their spatial extent, the inherent resolution in the data domain is limited despite the relatively high modeling frequencies. Most of the anomalies appear as blobs covering large areas, with less well-defined structures due to the diffusive nature of EM fields. However, the missing detailed features regarding production in Approach 1 can be picked up by Approach 2. This is when inversion needs to be carried out, which fits the data with the best model and leads us to Approach 2. Approach 2 is much more time-consuming than Approach 1 since inversion is involved, but it can provide more details caused by the oil-field production (Figure 14). We need to combine the two approaches for the best outcome regarding oil-field monitoring for production and safety. Ideally, future research should involve real-field time-lapse data during production to verify the credibility of the results in this paper.

The seismic method is one of the most effective monitoring methods. However, several studies have demonstrated that time-lapse seismic data struggle to discriminate between fluid saturation and pore pressure changes [17,48]. On the other hand, the time-lapse CSEM method can be very useful in reservoir production monitoring, because of its high sensitivity to pore fluid saturation and the effects of pressure changes being negligible compared to seismic data [19]. Thus, time-lapse CSEM is a complementary method to the seismic method in hydrocarbon production and CO_2_ storage monitoring.

A concern that needs to be mentioned in the context of marine 3D CSEM time-lapse monitoring repeatability is the existing infrastructure in the area and its implications on time-lapse signatures from hydrocarbon reservoirs and CO2 plumes [20]. Complex metallic structures can introduce serious challenges to the imaging and interpretation of subsurface resistivity alterations. These metallic structures include pipeline networks, well casings and subsea production templates. A study in 2010 revealed the contamination of CSEM data by pipeline interference [49]. Park et al. and Morten et al. [15,50] suggested different ways of circumventing and dealing with pipeline interference. One suggested approach [13] elaborates on the fact that the pipelines themselves can be used as secondary EM sources. To thoroughly understand the pipeline influence on the CSEM data, detailed and realistic modeling and analysis of the EM responses from complex pipeline networks will be necessary [51]. Therefore, the interplay between survey repeatability, high background resistivity and shallow reservoir burial depth at Wisting, in addition to potential metallic infrastructure on site would be interesting areas to investigate in future work.

## 6. Conclusions

In this paper, a technically feasible reservoir monitoring workflow suitable for time-lapse CSEM is presented. The workflow demonstrates that the CSEM experiment can be applied as a reservoir monitoring tool to enhance safe and effective production. Our results show that monitoring changes in the fluid saturation of realistic and detailed producing reservoirs can be captured by CSEM data, and this information can be further used in field development decisions.

We show how dynamic reservoir simulations related to different time instances can be converted to resistivity spatial distributions through a detailed, realistic, and geologically consistent rock-physics model. A modified Archie’s law is used and incorporated with available petrophysical information, developing a detailed rock physics model across the studied field. Using this rock physics model, water saturation models representing a specific production strategy through time are transformed into corresponding resistivity distribution maps. The constructed models are also calibrated with well data and available resistivity models. The presented production strategy spans from the year 2027 to 2057. This simulates that the hydrocarbon saturation decreases with time along with the bulk resistivity of the reservoir, which allows us to investigate how these resistivity changes in the pore fluid are reflected in the CSEM data.

A combination of 3000 m offset and 30 Hz frequency results in the most pronounced time-lapse anomalies, considering it an optimal choice for acquiring marine CSEM time-lapse monitoring data at Wisting. Our results suggest that higher frequencies than typical CSEM frequencies, in our case 30 Hz, give better sensitivity to 4D production effects. 

In the simulated CSEM surveys, five different towlines are used, where ten seabed receivers are positioned along each towline, giving a total of 50 seabed receivers. Receiver inline and crossline spacings are 1 km and 1.5 km, respectively. Such a receiver grid is suitable for capturing the time-lapse effects at Wisting. Most of the subsurface production effects are captured by the inline configuration.

Our results show that both CSEM data and ATR maps can resolve production-induced responses for five-year time windows in subsurface resistivity relatively early during the lifetime of a producing field. ATR maps in the model domain show more distinct anomalous patterns. Since the cost of a CSEM survey is relatively low, CSEM time-lapse is a feasible and cost-effective monitoring tool for oil production that can be conducted for Wisting.

The presented workflow proves the feasibility of using time-lapse CSEM to carry out safe production monitoring. Our study shows that both marine CSEM data and ATR maps can resolve production-induced changes in the electric resistivity of realistic and detailed hydrocarbon reservoirs offshore. This ensures more credibility for establishing time-lapse CSEM as a robust reservoir production and carbon storage monitoring tool and developing a feasible CSEM time-lapse workflow in the coming future.

## Figures and Tables

**Table 1:** Summary table of the key Archie parameters that are used to construct and calibrate the presented resistivity mapping.

Parameter	Value
a is an empirical constant	1
Well	Wisting central well (7324/8-1)
Cementation exponent (m)	2
Saturation exponent (n)	Non-linear ranging between 2.1 and 2.5
Formation water resistivity (R_w_)	0.18 ohm-m
Water saturation cut-off	10%
Gas saturation cut-off	70%

## Data Availability

The simulated CSEM data are available upon request.
